# Numerical Analysis Method That Considers Weathering and Water-Softening Effects for the Slope Stability of Carbonaceous Mudstone

**DOI:** 10.3390/ijerph192114308

**Published:** 2022-11-02

**Authors:** Yeyang Fu, Zhaohui Liu, Ling Zeng, Qianfeng Gao, Jintao Luo, Xinhui Xiao

**Affiliations:** 1 School of Traffic & Transportation Engineering, Changsha University of Science & Technology, Changsha 410114, China; 2 School of Civil Engineering, Changsha University of Science & Technology, Changsha 410114, China; 3 College of Civil Engineering, Hunan University of Technology, Zhuzhou 412007, China

**Keywords:** slope, carbonaceous mudstone, weathering, wetting, numerical simulation, rainfall infiltration

## Abstract

The mechanical behavior of carbonaceous mudstone deteriorates greatly when exposed to wet environments, and the precise evaluation of its slope stability has been a difficulty. This study aims to establish a numerical analysis method for the instability problems of its slopes; this method considers the effects of weathering and water-softening by establishing their mathematical expressions. The weathering and water-softening effects are reflected by variations in the mechanical properties (e.g., elastic modulus, angle of internal friction, and cohesion) of carbonaceous mudstone, with the depth following a logistic function and the shear strength parameters varying with wetting duration and degree of saturation. Their weathering and water-softening effects are reproduced with the use of the ABAQUS finite-element software and MATLAB programming. The proposed numerical method is applied to analyze the seepage field and stability of a highway cut slope with and without protection structures; the application results show that the proposed numerical method is reliable in analyzing the slope’s instability problem. The use of the herringbone skeleton structures can reduce the water-softening effects and thus increase the safety factor of the slope. The findings of this study could provide guidance to the design and construction of highway cut slopes in mountain areas that are rich in carbonaceous mudstone.

## 1. Introduction

Over the past decades, a great number of highway slopes have been created in southwestern China because of the sustained investment in transport infrastructure by the Chinese government. Carbonaceous mudstone is widely distributed in this mountain area. It has poor mechanical properties, such as easy weathering, low strength, and large deformation upon wetting, and it is a cause of environmental pollution after disintegration [1,2]. As a result, carbonaceous mudstone-related geological disasters such as slope instability frequently occur during rainfall seasons, which causes significant economic losses and serious environmental pollution. A major reason for these problems is the lack of reliable stability analysis methods for carbonaceous mudstone slopes. Therefore, it is essential to develop a stability analysis method that considers the above unfavorable properties, such as the weathering and water-softening of slopes.

In practical engineering, the physical and mechanical properties of soft rock have been observed to deteriorate due to weathering and rainwater softening [3,4,5,6]. Yu et al. [7] developed a multi-scale model to study the damage process of soft-rock slopes due to the change in rock microstructure with time. The model was based on a discrete element model and examined the effects of microfracture and pore development on the stability of soft rock slopes. Bhattarai et al. [8] as well as Nong and Towhata [9] reported that the shear strength and Young’s modulus of soft rocks gradually decrease as the weathering period increases, as determined by unconfined compression tests and triaxial tests. Qureshi et al. [10], Hachinohe et al. [11], and Lee and Yoon [12] investigated the weathering effect on exposed soft rocks with varying degrees of strength and durability, and they calculated the strength deterioration parameter for damaged rocks with different degrees of weathering based on wave velocity. Based on the classical elastoplastic theory and Perzyna’s overstress viscoplastic theory, Huang et al. [13] developed an elastoplastic and viscoplastic damage constitutive model for the short-term and long-term expansion and fracture behavior of soft rock extrusion deformation. Zhou et al. [14] analyzed the pore structure evolution characteristics of red-bedded soft rock in periodic water-rock interactions based on scanning electron microscopy (SEM) and digital image processing techniques. The deterioration of the soft rock mesostructure was found to be mainly reflected in the changes in pore shape and size. Rezaei et al. [15] investigated the uniaxial compressive strength, elastic modulus, water absorption, and other properties of diatomite under various drying and water-filled cycles. The authors described the degree of deterioration of various mechanical properties in terms of longitudinal wave velocity. Shakoor and Barefield [16] and Fu et al. [17] respectively conducted numerical studies and indoor experimental studies of the uniaxial compressive strengths of sandstone with various degrees of saturation. It was found that the peak strength and elastic modulus decreased as a negative exponential function with an increasing degree of saturation. Although there have been many studies on soft rock slopes, the mathematical equations for the weathering and water-softening of carbonaceous mudstone still need to be clarified.

The problem of slope instability caused by rainfall is a great concern in mountain areas [18,19]. Fu et al. [20] and Jiang et al. [21] used model tests and numerical simulations to investigate the effect of rainfall infiltration on the instability of slopes. The authors stated that rainfall significantly reduced slope stability. Ma et al. [22] performed finite element seepage calculations on strongly weathered slopes, and the results of their study showed that the depth of rainfall infiltration is proportional to the thickness of the soil layer. The work of Miscevic et al. [23] and Admassu et al. [24] showed that the overall stability of slopes is adversely correlated with the rate of weathering and the depth of weathering, and thus the weathering effect of the rock must be considered when analyzing slope stability. Li et al. [25] and Lin et al. [26] excavated high slopes of weathered soft rock and evaluated the factors affecting the excavation of weathered rock. Then, reinforcement measures were simulated, and it was found that the reinforcement was effective for slope instability. Bai et al. [27] obtained the characteristics of the whole process of flow-slip damage caused by granite residual soil landslide through indoor model tests. It was reported that the transport of loose material by surface runoff is an important reason for the prominent siltation in disaster-prone areas. Xu et al. [28], Park and Michalowski [29], and Ng et al. [30] conducted numerical analyses on the stability and failure patterns of slopes under rainfall infiltration. However, because of the differences in wetting nature between carbonaceous mudstone and other rocks, the above numerical methods cannot be directly used to analyze the stability of carbonaceous mudstone slopes.

The purpose of this study is to develop a numerical analysis method for the stability problem of carbonaceous mudstone slopes; this method considers weathering and water-softening effects. The mathematical expressions for the weathering and water-softening of carbonaceous mudstone are first established. The weathering and water-softening of carbonaceous mudstone are then reproduced with the ABAQUS finite element software incorporated in MATLAB programming. Finally, the reliability of the proposed numerical method is verified via an application in order to analyze the seepage field and stability of a highway cut slope under rainfall infiltration.

## 2. Methodology

### 2.1. Weathering Effect

**Figure 1:**
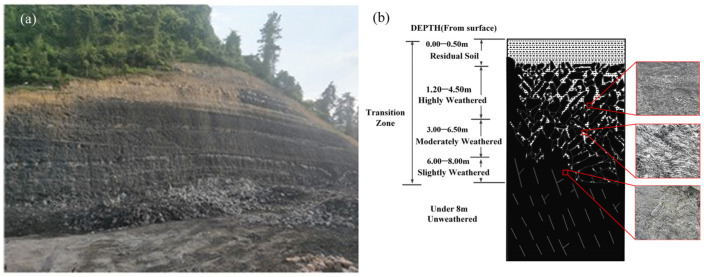
Weathering of carbonaceous mudstone: (**a**) photo; (**b**) schematic diagram.

The earth’s surface is subjected to a series of physical, chemical, and biological weathering effects. Generally, the closer the rock mass is to the earth surface, the stronger its weathering degree. Figure 1 shows the influence of weathering on carbonaceous mudstone in an excavated highway cut slope [2].

Because of the weathering effect, the mechanical properties of carbonaceous mudstone degrade as the depth decreases. The relationship between the mechanical properties and depth is often nonlinear. For this reason, it is assumed that the mechanical properties of carbonaceous mudstone (e.g., elastic modulus, angle of internal friction, and cohesion) vary with depth following a logistic function, which can be expressed by the equation below:

(1) $$\varpi =\frac{\sigma -\sigma_{\min}}{\sigma_{\max}-\sigma_{\min}}=\frac{1}{1+e^{-k^{*}(h-\frac{h_{0}}{2})}}$$

where ϖ is the coefficient of rock integrity (ϖ = 0–1) on the earth surface; σ, σ_min_, and σ_max_ are the mechanical property (e.g., elastic modulus, angle of internal friction, and cohesion) at a depth of *h* from the earth surface, the minimum mechanical property, and the maximum mechanical property, respectively; k^*^ is the slope of the rock weathering curve; *h*_0_ is the weathering depth of the rock.

Unlike the mechanical properties, the coefficient of permeability increases as the depth decreases. Thus, the relationship between the coefficient of permeability and the depth is expressed by a different equation:

(2) $$\varpi =\frac{\log k_{\max}-\log k}{\log k_{\max}-\log k_{\min}}=\frac{1}{1+e^{-k^{*}(h-\frac{h_{0}}{2})}}$$

where *k* is the coefficient of permeability; *k*_min_ and *k*_max_ are the minimum and maximum coefficients of permeability, respectively.

**Figure 2:**
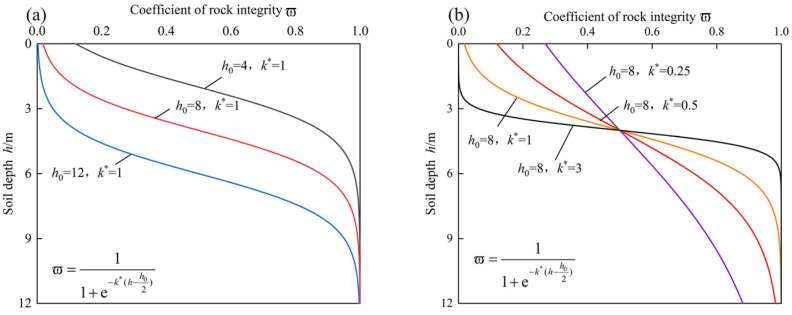
Relationship between the coefficient of rock integrity and depth: (**a**) effect of *h*_0_; (**b**) effect of k^*^.

Figure 2 depicts the effects of the parameters k^*^ and *h*_0_ on the logistic curves (the coefficient of rock integrity versus depth curves). The curves are mostly shifted up and down as the *h*_0_ value changes. The slope of the curve is controlled by k^*^. A large k^*^ indicates a sudden change in rock properties as the depth increases, whereas a small k^*^ shows a gentle change in rock properties as the depth increases. According to field research, the weathering depth of carbonaceous mudstone slopes is large, and the weathering degree weakens gradually as the depth increases; thus, *h*_0_ = 6–10 m and k^*^ = 0.5–1.0 are recommended.

### 2.2. Water-Softening Effect

Carbonaceous mudstone easily expands and disintegrates when exposed to rainwater as it contains numerous hydrophilic clay minerals, which inevitably cause the reduction of interparticle bonding and friction. In other words, the shear strength parameters, i.e., the cohesion and angle of internal friction, will decrease with an increase in wetting duration or degree of saturation under rainfall. The water-softening behavior of carbonaceous mudstone is assumed to follow Equation (3):

(3) $$\zeta =\frac{\delta -\delta_{{}^{w}}}{\delta }=A\left(1-e^{-bt}\right)\cdot \Delta S^{c}$$

where ζ is the rock water-softening coefficient (ζ = 0–1); δ is the cohesion or angle of internal friction; δ_w_ is the cohesion or angle of internal friction after water-softening; *t* is the wetting duration; Δ*S* is the relative degree of saturation, with Δ*S* = *S* − *S*_d_ (*S* is the degree of saturation and *S*_d_ is the degree of saturation in the natural dry state before wetting); *A*, b, and c are model parameters.

**Figure 3:**
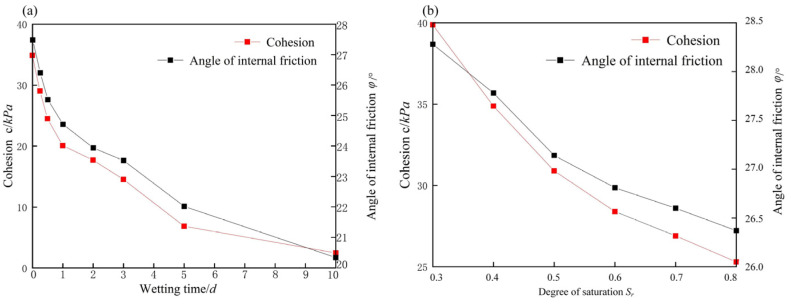
Effect of water-softening on the shear strength parameters of carbonaceous mudstone: (**a**) wetting duration (*S* = 1.0); (**b**) degree of saturation (*t* = 1 d).

**Figure 4:**
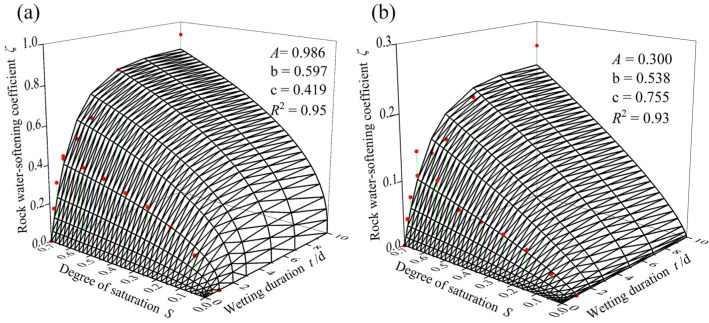
Fitting results of shear strength parameters of carbonaceous mudstone: (**a**) cohesion; (**b**) angle of internal friction.

Direct shear tests were conducted on weathered carbonaceous mudstone at different wetting durations and degrees of saturation in order to evaluate the effectiveness of Equation (3) and determine the model parameters. The test procedures were referred to the Chinese technical code (JTG 3430-2020), and the results are presented in Figure 3. It shows that with increasing wetting duration or degree of saturation, both the cohesion and the angle of internal friction of carbonaceous mudstone show a monotonic decreasing trend. The test data are fitted with Equation (3), as shown in Figure 4. The good fit indicates that Equation (3) can predict the water-softening behavior of carbonaceous mudstone. The model parameters are *A* = 0.986, b = 0.597, and c = 0.419 when the equation is used to predict the cohesion, while they are *A* = 0.300, b = 0.538, and c = 0.755 when it is used to predict the angle of internal friction.

### 2.3. Numerical Realization

The above analysis has shown that weathering and water-softening will deteriorate the engineering properties of carbonaceous mudstone, which inevitably increases the possibility of slope instability. Therefore, it is necessary to consider the combined effect of weathering and water-softening when analyzing the stability of carbonaceous mudstone slopes; otherwise, the calculated results may significantly deviate from the actual condition.

**Figure 5:**
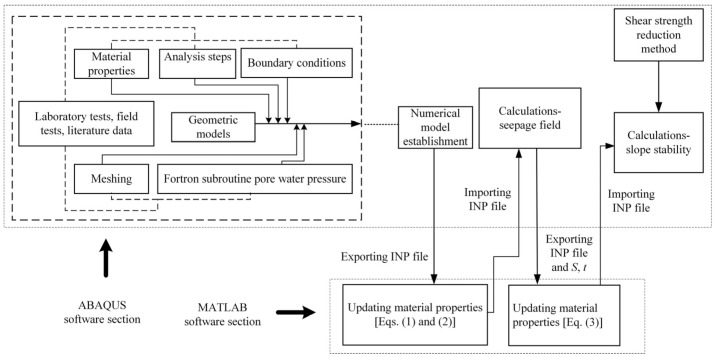
Realization of weathering and water-softening effects of carbonaceous mudstone in the finite element software.

Based on the ABAQUS software (Dassault Systemes, France), the numerical realization method to simulate the effect of weathering and water-softening on the behavior of carbonaceous mudstone is proposed for the slope stability analysis. The detailed procedures are as follows (Figure 5):

(1) The numerical model that considers rainfall infiltration is established. A geometric model of a carbonaceous mudstone slope is created in the ABAQUS software. Then, the material properties without considering the weathering and water-softening effects are assigned. After setting the proper meshes, analysis steps, and boundary conditions (e.g., gravity, rainfall boundary, and initial profile of pore water pressure), the numerical model is established.
(2) The ABAQUS Input (INP) file is modified to simulate the weathering effect. The depth of each element relative to the original ground surface in the slope is determined by MATLAB programming after the INP file is exported from the ABAQUS software. Then, the material properties of carbonaceous mudstone at various depths after weathering are calculated by Equations (1) and (2), and the weathering effect is taken into account by updating the material properties of every element in the INP file.
(3) The seepage field of the slope under rainfall infiltration is calculated. Numerical calculation is conducted in the ABAQUS software using the modified INP file. After the finite element analysis, the seepage data of the slope, including the degree of saturation and wetting duration of every element, are determined.
(4) The INP file is again modified to consider the water-softening effect. Based on the degree of saturation and the wetting duration of each element in the slope, MATLAB programming is employed to calculate the shear strength parameters of carbonaceous mudstone after water-softening following Equation (3). The material properties of every carbonaceous mudstone element in the INP file are updated for the second time to simulate the water-softening effect.
(5) The stability of the carbonaceous mudstone slope is calculated. The modified INP file is imported into the ABAQUS software, and then the stability of the carbonaceous mudstone slope is analyzed using the shear strength reduction method. Finally, the safety factor and the failure mode of the slope are determined.

### 2.4. Case Application

In this section, the above numerical method is applied to analyze the seepage behavior and stability of a carbonaceous mudstone cut slope before and after protection, respectively.

The research object is a cut slope on the Long-Lang expressway in Hunan Province, China. This region has a hilly landform and a subtropical monsoon climate, with abundant rainfall in Spring and Summer. According to the monitoring information from the meteorological department of Loudi City, Hunan Province, China, the annual average temperature is 16.5–17.5 °C, the annual average precipitation is 1300–1400 mm, and the annual evaporation is 1365.6–1521.6 mm. The maximum precipitation per day is 147.3 mm.

**Figure 6:**
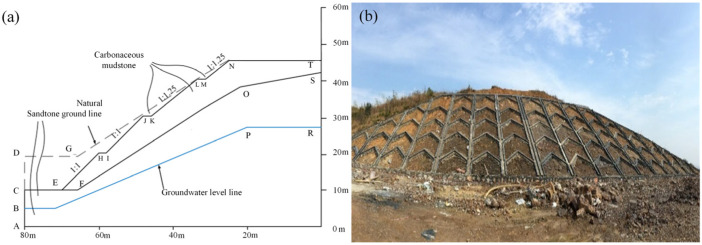
Basic information on the carbonaceous mudstone slope: (**a**) sketch; (**b**) photo.

The cut slope has a maximum height of about 35 m and is divided into four stages. The two lower stages and two higher stages have a rise/run ratio of 1:1 and 1:25, respectively. The heights of the first three stages are 10.0 m, and that of the highest stage is 5.0 m. There are platforms with widths of 2.0 m between every two stages. The upper layer of the slope is strongly weathered carbonaceous mudstone, while the lower layer is medium-weathered sandstone. Figure 6a depicts a representative cross section of the studied slope, with its material types and groundwater level indicated. The dashed line is the natural ground of this slope before excavation. Herringbone concrete skeletons have been designed to protect this carbonaceous mudstone slope as they can serve as intercepting channels that drain runoff from the slope surface. Figure 6b is a photo of the slope taken in the construction phase.

### 2.5. Numerical Model

#### 2.5.1. Geometric Model and Meshing

**Figure 7:**
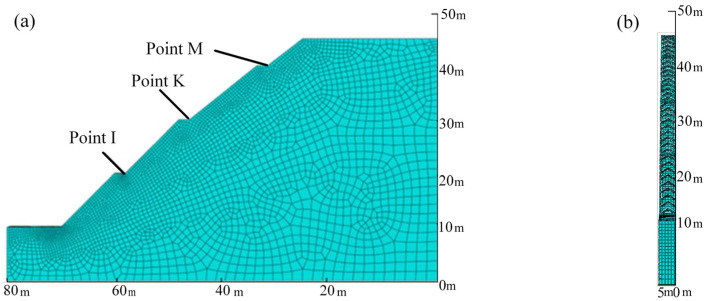
Numerical model of the studied slope: (**a**) lateral view; (**b**) front view.

A three-dimensional numerical model was created. The length and height of the model is 80 m and 45 m, respectively. The angle of the first and second steps here is 45°, and the angle of the third and fourth steps is 33°. To reduce calculation costs, only one protective structure cell was considered; thus, the width of the model is 5.0 m. After meshing, a total of 26,865 elements and 27,850 nodes were generated for the numerical model. The slope model was simulated with C3D8R elements, which are 8-node linear brick elements with reduced integration. The protection structure was simulated with C3D10 elements, which are 10-node tetrahedral. The meshed numerical model is illustrated in Figure 7.

#### 2.5.2. Material Properties

**Figure 8:**
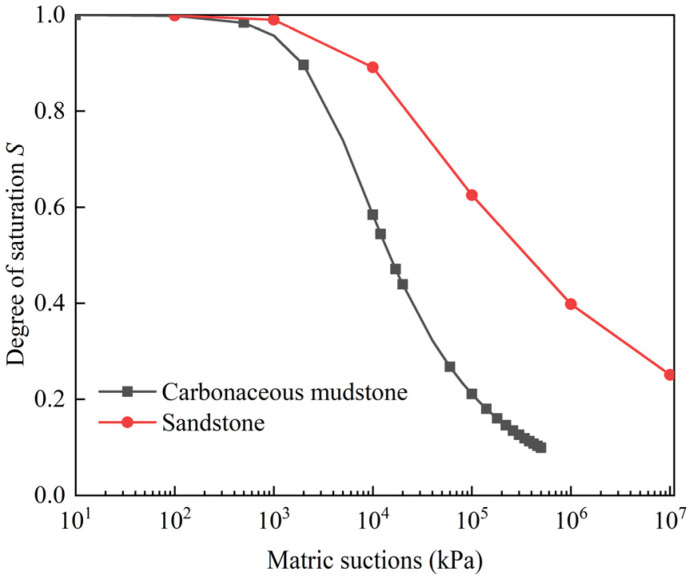
Soil–water characteristic curve.

Table 1 shows the physical, mechanical, and hydraulic properties of the materials determined by a series of laboratory and in situ tests following the Chinese technical codes (JTG E41-2005; JTG 3430-2020). For some properties, value ranges are provided, which indicate that these properties vary with depth because of the weathering effect. The variation between the limit values is expressed by Equation (1) or (2). The soil–water characteristic curves that characterize the relationship between the degree of saturation and the matric suction of the materials were measured using the pressure plate method [31], as shown in Figure 8.

#### 2.5.3. Boundary Conditions

To consider the initial unsaturated conditions of the slope, the linear distribution of negative pore water pressure was imposed on the slope along the vertical direction. The negative pore water pressure increased from zero at the groundwater level to the maximum at the slope surface. The nodes at the bottom boundary of the numerical model were fully fixed in both the vertical and horizontal directions, and the nodes at the lateral boundary were fixed in the horizontal direction. The top surface of the slope model, excluding protection structures, was considered to be a rainfall boundary. Based on the meteorological data of the studied region, a rainfall intensity of 0.1 m/d and a rainfall duration of 10 d were used in the numerical simulation. A rainfall event with this intensity is considered to be extremely heavy rainfall according to the Chinese rainfall intensity classification system.

## 3. Results and Discussion

### 3.1. Slope Behavior before Protection Structures

**Figure 9:**
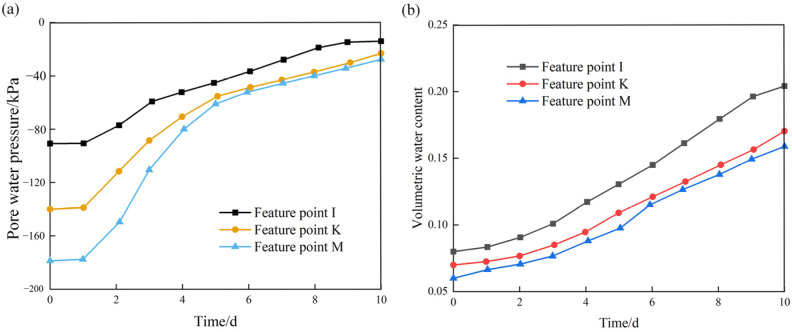
Variations in pore water pressure and volumetric water content with time at three positions on the slope without protection: (**a**) pore water pressure; (**b**) volumetric water content.

Figure 9 plots the variations in pore water pressure and volumetric water content with time at three representative points (I, K, and M, see Figure 7) on the slope without protection during rainfall infiltration. It can be seen that with an increase in rainfall time, both the pore water pressure and volumetric water content show a monotonic increasing tendency. In particular, the pore water pressure rises rapidly from the third day to the fifth day, and then the rising trend slows down until the end of rainfall. By contrast, the volumetric water content continues to increase rapidly for the whole rainfall duration.

**Figure 10:**
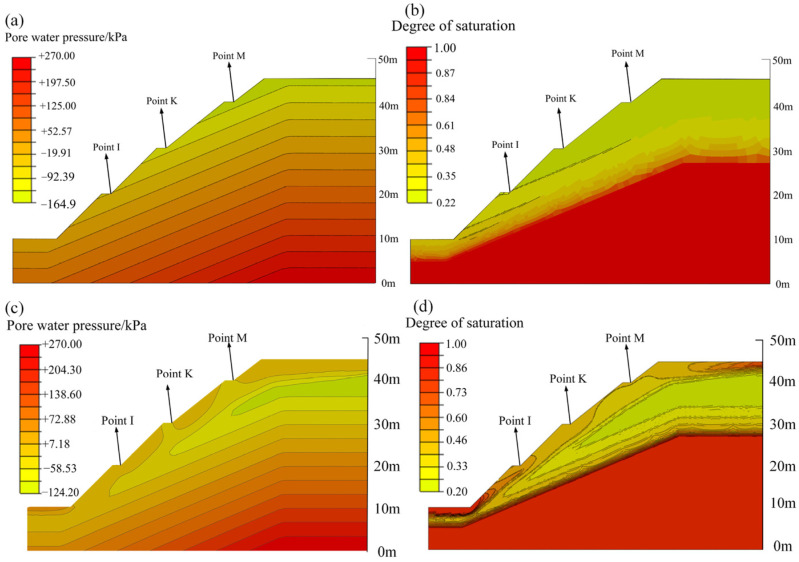
Contour maps of infiltration characteristics of the slope without protection: (**a**) pore water pressure before rainfall; (**b**) degree of saturation before rainfall; (**c**) pore water pressure after rainfall; (**d**) degree of saturation after rainfall.

**Figure 11:**
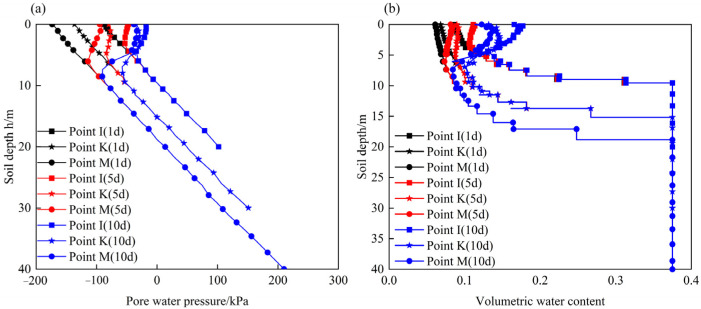
Distribution curves of pore water pressure and volumetric water content with the depth of the slope without protection after rainfall: (**a**) pore water pressure; (**b**) volumetric water content.

Figure 10 shows the contour maps of pore water pressure and the degree of saturation of the unprotected slope before and after rainfall. After a rainfall duration of 10 d, the shallow soil at the toe of the slope tends to be saturated. There is no transient saturated zone on the slope surface because the slope surface is highly weathered, and its permeability coefficient is greater than the rainfall intensity. At the same time, the degree of saturation within a certain depth below the slope surface and the crest of the slope increases significantly. This can be seen more clearly from the curves of the pore water pressure and the volumetric water content at different depths below the three representative points (I, K, and M) in Figure 11. The Figure shows that rainfall-caused increases in pore water pressure and volumetric water content occur at a depth of 4 m, 6 m, and 8 m below points I, K, and M, respectively. At these depths, the distributions of pore water pressure and volumetric water content follow the initial curves before rainfall. This is because the permeability coefficient of rock is reduced as the depth increases due to the weathering effect, and it is more difficult for rainwater to infiltrate downward.

**Figure 12:**
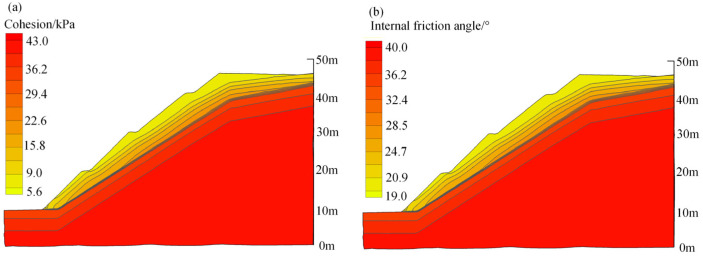
Contour maps of shear strength parameters that consider weathering and water-softening effects: (**a**) cohesion; (**b**) angle of internal friction.

**Figure 13:**
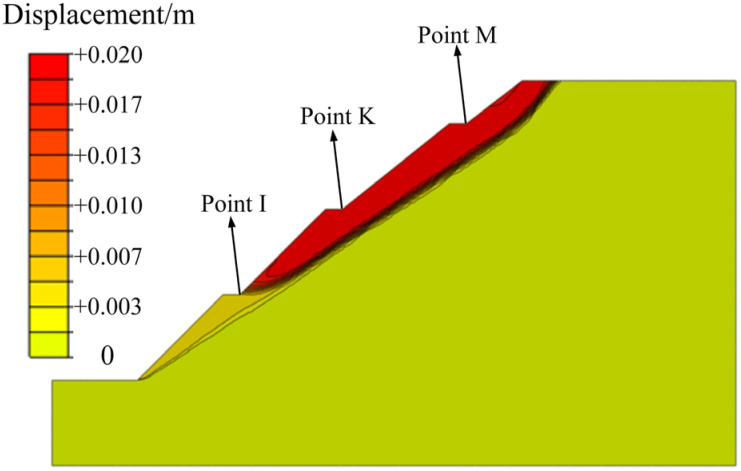
Failure mode of the slope without protection after rainfall infiltration.

After rainfall infiltration, the engineering properties of carbonaceous mudstone further deteriorated due to the water-softening effect. The contour maps of the shear strength parameters of the slope that considered both the weathering and water-softening effects are shown in Figure 12. After slope stability calculation, the results indicate that the safety factor of the slope without protection after rainfall infiltration is 1.55. Figure 13 plots the contour map of the displacement of the slope at the critical failure state. It can be noted that this carbonaceous mudstone slope shows a shallow-sliding failure mode. The sliding surface of the slope starts from the toe to the crest of the slope, but most of the unstable soil is above the second stage. The depth of the unstable soil is less than 6 m, which is consistent with the rainwater infiltration depth (Figure 11). This suggests that the reason why the carbonaceous mudstone slope shows shallow failure rather than deep circular failure is that the rock in the shallow layer of the slope is subjected to water-softening effects due to rainfall infiltration. To increase the stability of the carbonaceous mudstone slope, some protection measures that could reduce rainfall infiltration or improve drainage conditions are recommended.

### 3.2. Slope Behavior with Herringbone Concrete Skeleton Protection

**Figure 14:**
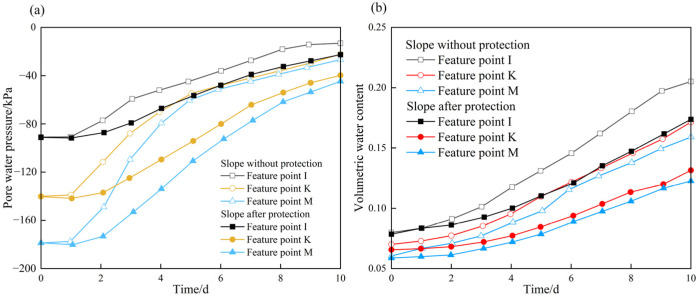
Variations in pore water pressure and volumetric water content with time at three positions on the protected slope: (**a**) Pore water pressure; (**b**) Volumetric water content.

The seepage field and stability of the carbonaceous mudstone slope after being protected with herringbone concrete skeletons are also analyzed. Figure 14 shows the changes in pore water pressure and volumetric water content with time at three representative points (I, K, and M) on the protected slope and unprotected slope during the rainfall period. It can be noted that the pore water pressure and volumetric water content show similar variation trends to those of the unprotected slope (Figure 9); however, the variations exhibit a delay, and the rising amplitudes are smaller after the slope is protected. For example, the pore water pressure of the unprotected slope tends to rise from the first day, while that of the protected slope rises from the second day. The maximum pore water pressure and volumetric water content at point I of the unprotected slope are −22.3 kPa and 0.18, respectively. Nevertheless, the maximum pore water pressure and volumetric water content at point I of the slope after protection are −13.99 kPa and 0.21, respectively.

**Figure 15:**
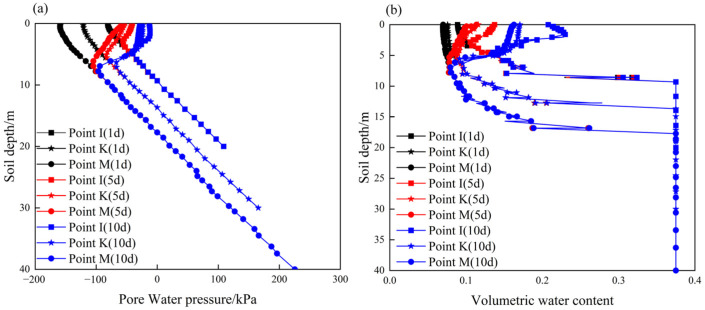
Distribution curves of pore water pressure and volumetric water content with depth of the protected slope after rainfall: (**a**) pore water pressure; (**b**) volumetric water content.

After rainfall, the contour maps of the pore water pressure and the degree of saturation of the protected slope are broadly similar to those of the unprotected slope. However, some differences in values can be observed. Figure 15 illustrates the changes in pore water pressure and volumetric water content with depth on the protected slope. One can note that both the maximum pore water pressure and volumetric water content of the shallow layer of the protected slope are smaller than those of the unprotected slope. For example, after being protected with a herringbone skeleton structure, the maximum pore water pressure and volumetric water content at point I of the slope are reduced by 59.43% and 15.03%, respectively. The above results indicate that the existence of a protective structure can reduce rainfall infiltration.

**Figure 16:**
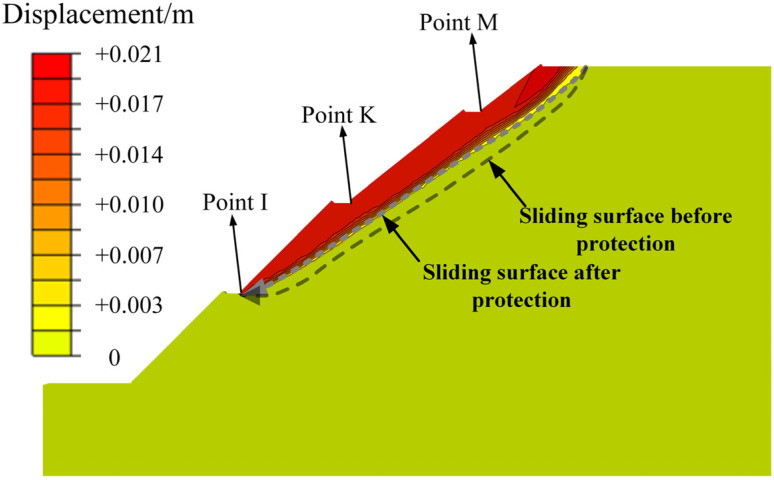
Failure mode of the protected slope after rainfall infiltration.

A stability analysis showed that the safety factor of the carbonaceous mudstone slope improved from 1.55 to 1.65 after protection. Figure 16 plots the contour map of displacement of the protected slope at the critical failure state. When the slope was protected with the herringbone skeleton structure, the area of the unstable soil became obviously smaller, and the thickness of the unstable soil was reduced. Moreover, the failure mode of the carbonaceous mudstone slope was slightly changed because of the slope protection. Unlike the previous case (Figure 13), the new sliding surface starts from the foot of the second stage and continues to the crest of the slope. This means that the protective structure is particularly conducive to the stability of the first slope stage. This finding is consistent with the above results, proving that the herringbone skeleton structure is able to retard rainfall infiltration and thus reduce the wetting-softening effect. Therefore, the use of the herringbone skeleton structure is effective in protecting carbonaceous mudstone slopes.

## 4. Conclusions

This study developed a numerical analysis method for the stability problem of carbonaceous mudstone slopes; the method considered both weathering and water-softening effects. The reliability of the proposed method was verified via an application to analyze the seepage field and stability of a carbonaceous mudstone cut slope under rainfall infiltration. The conclusions are as follows:

(1) The relationship between the mechanical and hydraulic properties of carbonaceous mudstone and the depth on a slope is assumed to be a logistic function. On this basis, a mathematical equation was established to consider the effect of weathering on carbonaceous mudstone slopes.
(2) Laboratory tests showed that the shear strength parameters were reduced, following an exponential form, as the wetting duration increased; at the same time, they showed a decreasing trend as the degree of saturation increased. Therefore, the rock water-softening coefficient that was a function of wetting duration and degree of saturation was introduced, which could characterize the effect of water-softening on carbonaceous mudstone.
(3) The consideration of the effects of weathering and water-softening on carbonaceous mudstone in the ABAQUS finite element software was realized by updating the INP file. The material properties in the INP file were modified by MATLAB programming based on the mathematical equations of weathering and water-softening.
(4) The above proposed numerical analysis method was used to analyze the seepage field and stability of a highway cut slope with and without herringbone concrete skeletons being subjected to rainfall. The results indicated that the use of herringbone concrete skeletons can retard rainfall infiltration and enhance the stability of carbonaceous mudstone slopes.

In this study, the weathering effect was assumed to regularly change with depth, and thus material variability at the same depth was neglected. This assumption may have caused differences between the calculated seepage field and the actual situation, causing some errors in the safety factor of the slope. Therefore, future work could take weathering variability into consideration when analyzing the behavior of carbonaceous mudstone slopes.

## Figures and Tables

**Table 1:** Material properties of the studied slope.

Material	Density (kg/m^3^)	Cohesion (kPa)	Angle of Internal Friction (°)	Elastic Modulus (MPa)	Poisson’s Ratio	Void Ratio	Permeability Coefficient (m/d)
Carbonaceous mudstone	2000	22.5–42.57	21.4–28.24	120–750	0.33	0.6	0.864–0.0864
Sandstone	2250	41.5–43.0	27.6–40.0	800–1150	0.23	0.6	0.02592–0.0002592

## Data Availability

Data available from the first author.
